# DHCR24 reverses Alzheimer’s disease-related pathology and cognitive impairment via increasing hippocampal cholesterol levels in 5xFAD mice

**DOI:** 10.1186/s40478-023-01593-y

**Published:** 2023-06-21

**Authors:** Wen-bin Zhang, Yue Huang, Xiao-rou Guo, Meng-qi Zhang, Xiang-shan Yuan, Heng-bing Zu

**Affiliations:** 1 Department of Neurology, Jinshan Hospital Affiliated to Fudan University, No.1508 Long-Hang Road, Jinshan District, Shanghai, 201508 China; 2 Department of Anatomy and Histoembryology, School of Basic Medical Sciences, Fudan University, Shanghai, 200032 China; 3 State Key Laboratory of Medical Neurobiology and Ministry of Education Frontiers Center for Brain Science, Institutes of Brain Science, Fudan University, Shanghai, 200032 China

**Keywords:** Alzheimer’s disease, 24-dehydrocholesterol reductase (DHCR24), Cholesterol, Gene therapy, Neuroprotection, Neurodegeneration, Pathogenesis

## Abstract

Accumulating evidences reveal that cellular cholesterol deficiency could trigger the onset of Alzheimer’s disease (AD). As a key regulator, 24-dehydrocholesterol reductase (DHCR24) controls cellular cholesterol homeostasis, which was found to be downregulated in AD vulnerable regions and involved in AD-related pathological activities. However, DHCR24 as a potential therapeutic target for AD remains to be identified. In present study, we demonstrated the role of DHCR24 in AD by employing delivery of adeno-associated virus carrying DHCR24 gene into the hippocampus of 5xFAD mice. Here, we found that 5xFAD mice had lower levels of cholesterol and DHCR24 expression, and the cholesterol loss was alleviated by DHCR24 overexpression. Surprisingly, the cognitive impairment of 5xFAD mice was significantly reversed after DHCR24-based gene therapy. Moreover, we revealed that DHCR24 knock-in successfully prevented or reversed AD-related pathology in 5xFAD mice, including amyloid-β deposition, synaptic injuries, autophagy, reactive astrocytosis, microglial phagocytosis and apoptosis. In conclusion, our results firstly demonstrated that the potential value of DHCR24-mediated regulation of cellular cholesterol level as a promising treatment for AD.

**Supplementary Information:**

The online version contains supplementary material available at 10.1186/s40478-023-01593-y.

## Introduction

So far, the amyloid hypothesis has dominated the research on Alzheimer’s disease (AD) [27, 75]. However, the repeated failures of clinical trials targeting anti-amyloid-beta (Aβ) have challenged our narrow understanding of AD pathogenesis, suggesting Aβ could be a prominent pathological feature but not exclusive causative factor, and inspiring wide-ranging investigations into the underlying mechanisms of AD [9, 32, 55, 64, 73, 77]. Although a number of hypotheses about the cause of AD have been proposed, the ultimate etiology of AD remains elusive [5, 6, 14, 27]. Because no effective treatments are still available for AD, the development of new disease-modifying treatment could become a really urgent need.

Interestingly, a tight link between AD neuropathology and lipid was proposed by Alois Alzheimer, who described “lipoid granules” as a third neuropathological hallmark of AD [26]. Since the early 1990s, many subjects have focused on the relationship between cholesterol metabolism and AD, including biology, epidemiology, and genetics [6, 10, 26, 47]. Although a few of researches thought the high level of serum cholesterol was a hazard factor for AD, but it was ignored that the synthesis and supply or metabolism of cholesterol was independent in brain because of blood–brain barrier [6, 47, 61]. Thus, these evidences do not support the correlations between high plasma cholesterol and pathology of AD [6, 26, 47, 61]. Surprisingly, statins played beneficial role in many diseases by lowering serum cholesterol levels, but many studies also suggested it may show adverse effects due to the decrease of brain cholesterol levels [12, 45]. What’s more, Tamoxifen, which induced cognitive impairment in cancer therapy, was also found lead to cellular stress via inhibiting cholesterol synthesis [17].

Furthermore, accumulating data indicate a tight correlation between brain cholesterol deficiency and AD pathogenesis, which means forming a new cholesterol hypothesis [6, 10, 23, 47]. A growing body of evidences showed lower brain cholesterol, coupled with decreased cholesterol synthesis and trafficking in AD patients and animals, along with aging human and animals [6, 10, 35, 47, 52, 56, 57, 66, 83]. In addition, mutation or polymorphism of genes involving in brain cholesterol trafficking, including apolipoprotein E4 (APOE4), ATP binding cassette (ABC), low-density lipoprotein receptor (LDLR) family, and Niemann-Pick type C1/2 (NPC1/2), lead to reduced cholesterol efflux and transport and impaired cholesterol influx into neurons, eventually resulted in neuronal cholesterol deficiency, which could be tightly related to AD pathology, such as Aβ deposition, phosphorylated tau accumulation, reactive astrocytosis, microglial phagocytosis, apoptosis and synaptic injuries [6, 10, 21, 42, 48, 71, 74].

What’s more, it was identified that 24-dehydrocholesterol reductase (DHCR24), a key regulator of synthesis and metabolism of cholesterol, was downregulated in vulnerable regions in AD patient brain [25, 31]. In previous vitro studies, we demonstrated a loss in membrane and intracellular cholesterol by knocking down DHCR24, coupled with disruption of membrane lipid raft and abnormality of raft-dependent cell signaling, could leads to Aβ generation, tau hyperphosphorylation, synaptic injuries and inflammation [6, 7, 49]. As a key enzyme in the biosynthesis of cholesterol, DHCR24 controlled cellular cholesterol synthesis and homeostasis [6]. Genetic evidences and outcomes from cell models strongly support cellular cholesterol deficiency contributes to AD pathogenesis [6, 10]. Therefore, compelling evidences support a new hypothesis that brain cellular cholesterol loss could trigger the onset of AD.

To date, more and more therapy research focused on the cholesterol is coming to our attention. A possible treatment of AD of cholesterol-based gene therapy has been studied in many preclinical trials displaying inconsistent results [8, 29]. However, accumulating data suggests that ferrying the genes that are involved in cholesterol metabolism into mice brain could affect AD-related pathology and cognitive activity [8, 22, 29, 76]. Notably, in previous vitro studies, we demonstrated that DHCR24 knock-in could alleviate AD-related pathological impairments by modulating the level of cellular cholesterol, including Aβ generation, tau hyperphosphorylation, synaptic injuries, autophagy and apoptosis [6, 7, 15, 43, 49, 63]. As a key enzyme, DHCR24 could play a neuroprotective role in AD-related pathology by controlling cellular cholesterol synthesis and homeostasis [6]. In present study, we demonstrated the effect of DHCR24 in AD by AAV-mediated DHCR24 gene delivery in 5xFAD mice. Surprisingly, the cognitive impairment and AD-related pathology of 5xFAD mice was significantly reversed after DHCR24 knock-in. Taken together, based on the hypothesis of brain cellular cholesterol deficiency in AD, increasing evidences suggest that modifying brain cellular cholesterol metabolism by DHCR24 could provide a potential treatment for AD.

## Materials and methods

### Animals

5xFAD mice (male, 3 months, weight 17–23 g, Jackson Laboratory, 034848-JAX) and wild-type (WT) littermates were used in this experiment. The transgenic mice over-expressed both mutant human amyloid precursor protein (APP) and PS1 genes, which develop amyloid plaques and have a decline in long-term spatial working memory in 2 or 3 months, but not display neurofibrillary tangles [19]. With enough food or water, all mice were kept at the temperature of 22 °C in a 12–12 h light/dark cycle (lights on at 7 a.m. to 7 p.m., light intensity: 100 lx). Efforts were made into minimize animals used along with any pain experienced by mice as possible. All procedures and experiments were performed according to guidelines authorized by Animal Experiment and Use Committee at Shanghai Medical College of Fudan University (Permit No. 20210302-026).

### AAV injection and stereotaxic surgery

DHCR24 overexpression virus (AAV2/9-ZsGreen-DHCR24) and control virus (AAV2/9-ZsGreen), packaged and provided by Hanbio Tech (Shanghai, China), were injected into 5xFAD mice and WT mice (aged 3 months) under anesthesia of isoflurane via anesthesia mask. We used the following coordinates to inject AAV-DHCR24 cDNA or AAV-vector into bilateral hippocampus: − 2.3 mm anteroposterior and ± 1.8 mm mediolateral from the bregma, and − 1.2 mm dorsoventral from the surface of dura mater. Viral suspension (1 μL) containing 1.5 × 10^12^ vector genomes per mL was placed into the target area at a rate of 100 nL/min by Using the Nanoject II (Drummond Scientific, Broomall, PA). After the injection, the pipette was retained for 15 min to allow absorption of AAV before being withdrawn. And then, keeping mice in a heater about 30–60 min to keep them warm during surgery. The mice underwent Morris water maze (MWM) 3–4 weeks after stereotactic injection, and were sacrificed at 7 days after the MWM testing.

### Immunohistochemistry and immunofluorescence

One month after the AAV injection, the mice were anesthetized by using isoflurane via anesthesia mask. First, the transcardiac perfusion of mice was by 0.01 M phosphate-buffered saline (PBS) and then by 4% paraformaldehyde (PFA). Brains were carefully isolated and fixed in 4% PFA for 12–36 h at 4 °C, finally placed them in 30% sucrose for 48–72 h at 4 °C. All brains were prepared for frozen or paraffin sections according to experiments.

To the frozen sections, first brains should be embedded with OCT compound and be stored at − 80 °C, and then were coronally sectioned at 30 μm on a cryostat microtome (Leica 1950). The 30 μm brain slices were collected in 0.01 M PBS and then be washed with PBS for three times. And then, the brain slices were incubated with 3% donkey serum (v/v), 0.25% Triton (v/v) and primary antibodies at 4 °C overnight. Brain slices were then washed in PBS and incubated with Alexa 594 or Alexa 647 conjugated secondary antibodies (1:1000, Jackson ImmunoResearch) for 2 h in a room temperature (RT). Finally, brain slices were then washed again in PBS and cover-slipped with DAPI Fluoromount-G mounting medium (Southern Biotech, 0100-20). The fluorescence signals were detected by confocal microscope (Leica SP8, Germany) and VS120 virtual microscopy slide scanning system (Olympus), and the same parameters settings were employed between different groups.

To the paraffin sections, brains were embedded in paraffin wax, and cut into 4 microns-thick sections. After dewaxing, the brain slices were also incubated with 3% donkey serum (v/v), 0.25% Triton (v/v) and primary antibodies at 4 °C overnight. Then incubated with biotinylated IgG (1:1000, Jackson ImmunoResearch) for 2 h in RT, and then incubated with ABC complex for 2 h in RT. Next, sections incubated with DAB (0.2 mg/ml) for 10 min in RT, and then mounted onto slides, dehydrated, and coverslipped.

Primary antibodies were used as follows: DHCR24 (1:500; ab137845, abcam); Beta Amyloid [MOAB-2] (1:400; ab126649, abcam); Synapsin I (1:500; ab64581, abcam); GFAP (1:2000; ab4674, abcam); NeuN (1:1000; ab177487, abcam); Iba1 (1:1000; PRB029, OasisBiofarm); CD68 (1:200; ab955, abcam).

### Filipin III staining

Cholesterol level of hippocampus was evaluated by Filipin III staining. Brains embedded with OCT were coronally sectioned at 10 μm on a cryostat microtome, then mounted onto slides, and then fixed by 4% PFA for 10 min. Sections were incubated in 1.5% glycine in PBS for 10 min followed by washed with PBS for three times. After 5 min washes in PBS for three times, sections were incubated for 2 h at RT with Filipin (0.05 mg/ml; SAE0087, Sigma) in PBS. After this, sections were washed with PBS again and coverslipped with fluorescent mounting media (DakoCytomation). The fluorescence signals of Filipin were detected by confocal microscope (Leica SP8, Germany) and the same settings were employed between different groups.

### TUNEL staining

Apoptosis in the hippocampus was evaluated by TdT-mediated dUTP Nick-End Labeling (TUNEL) staining. Brains embedded with OCT were coronally sectioned at 10 μm on a cryostat microtome, then mounted onto slides. Subsequently, the brain slices were incubated in 0.5% Triton for 10–20 min and then washed in PBS. We used One Step TUNEL Apoptosis Assay Kit (C1090, Beyotime Biotechnology, China), 50 μL TUNEL reagent prepared as described in the instructions was added into each slice, and then slices were cultured in a dark environment at 37 °C for 2 h. After washing PBS as above, slices were cover-slipped with DAPI Fluoromount-G mounting medium (Southern Biotech, 0100-20). The fluorescence signals were detected by confocal microscope (Leica SP8, Germany) and the same settings were employed between different groups.

### Western blot

The animals were decapitated, separated hippocampus carefully, and then stored at − 80 °C. Extracts from hippocampus were obtained in cold SDS lysis buffer (Merk, Shanghai), supplemented with both phosphatase inhibitor (1:100, Millipore) and protease inhibitor (1:100, Millipore). After centrifuged about 15 min at 12,000 ×*g* (at 4 °C), the total protein concentration in tissue homogenates was detected by BCA assay kit (Thermo Scientific), and supernatants were then boiled for 5–10 min after mixing with loading buffer tricine (Takara). Subsequently, an equal amount (20 ug) of proteins was fractionated by SDS PAGE on 10% or 12.5% polyacrylamide gels (PG112 and PG113, EpiZyme Biotechnology, China) according to their concentrations. Afterwards, proteins were transferred onto 0.2 μm or 0.45 μm PVDF membranes (Millipore) and then blocked with 5% skimmed milk in TBS containing 0.1% Tween 20 (TBST) at RT for 1 h. Besides, the bands were incubated with primary antibodies at 4 °C overnight. After washing the bands 3–4 times in TBST, then incubating it for 1 h at RT with secondary antibodies (1:5000, Proteintech, China) in Secondary Antibody Dilution Buffer (Beyotime, China). Finally, after washing again with TBST 3–4 times, the bands were detected by Immobilon ECL Ultra Western HRP (Millipore) and detected by Tannon 4600 Chemiluminescence Image Analysis System version 3.0 (Tannon, Shanghai, China). All experiments had been repeated at least 3 times.

Primary antibodies involved are as follows: DHCR24 (1:1000; ab137845, abcam); APP (1:1000; 2452S, Cell Signaling); SREBP2 (1:500; ab30682, abcam); HMGCR (1:800; DF6518, Affinity); Phospho-mTOR (1:1000; 5536S, Cell Signaling); mTOR (1:1000; 2983S, Cell Signaling); Phospho-GSK-3β (1:1000; 9323S, Cell Signaling); GSK-3β (1:1000; 12456S, Cell Signaling); Beclin-1 (1:1000; 3495S, Cell Signaling); SQSTM1/p62 (1:1000; 39749S, Cell Signaling); LC3B (1:1000; L7543, Sigma); Phospho-p38 MAPK (1:1000; 4511S, Cell Signaling); p38 MAPK (1:1000; 8690S, Cell Signaling); Phospho-JNK(1:1000; 4668S, Cell Signaling); JNK (1:1000; 9252S, Cell Signaling); Bax (1:1000; 5023S, Cell Signaling); Bim (1:1000; 2933S, Cell Signaling); Bcl2 (1:2000; 26593-1-AP, Proteintech); Caspase-3 (1:500; 19677-1-AP, Proteintech); Phospho-Erk1/2 (1:1000; 4370S, Cell Signaling); Erk1/2 (1:1000; 4695S, Cell Signaling); Phospho-MEK1/2 (1:1000; 9154S, Cell Signaling); MEK1/2 (1:1000; 8727S, Cell Signaling); RhoA (1:1000; 2117S, Cell Signaling); PSD95 (1:1000; 3409S, Cell Signaling); Synapsin I (1:2000; ab64581, abcam); HRP-conjugated GAPDH (1:10000; HRP-60004, Proteintech).

### RT-PCR

Total RNA from hippocampus was isolated by the Total RNA extraction reagent RNAiso Plus (9108/9109, Takara, Japan). Reverse transcription used PrimeScript™ RT Master Mix (Takara, Japan) and PCR used TB Green Premix Ex (Takara, Japan). The threshold cycle (Ct) was acquired with Sequence Detection Software (Applied Biosystems, USA). The analysis of results was by ΔΔCt method and every target mRNA was normalized with the GAPDH. Primer sequences of DHCR24, HMGCR, SREBP2 and GAPDH were shown in Table 1.

**Table 1:** List of primers and primer sequences

Genes	Forward primer (5ʹ–3ʹ)	Reverse primer (5ʹ–3ʹ)
DHCR24	CGCTGCGAGTCGGAAAGTA	GTCACCTGACCCATAGACACC
HMGCR	ATGCCTTGTGATTGGAGTTG	GTTACGGGGTTTGGTTTATT
SREBP2	GCAGCAACGGGACCATTCT	CCCCATGACTAAGTCCTTCAACT
GAPDH	CTGCCCAGAACATCATCC	CTCAGATGCCTGCTTCAC

### Aβ ELISA

To detect the level of Aβ40 and Aβ42 in hippocampus, we separated the hippocampus and then stored at − 80 °C. Extracts from hippocampus were obtained in cold SDS lysis buffer (Merk, Shanghai), supplemented with protease inhibitor (1:100, Millipore). After centrifuged for 15 min at 12000 ×*g* (at 4 °C), the total protein concentration in tissue homogenates was determined using a BCA protein assay kit (Thermo Scientific). And then, the isolated protein samples were transferred to Aβ40 and Aβ42 ELISA kits (27,720 and 27,721, IBL, Japan), the measurement was according to protocol recommended by manufacturer. Most notably, the amount of Aβ40 and Aβ42 should were normalized with total protein concentration by BCA assay kits. These experiments duplicated at least 3 times and the data were acquired from independent experiments.

### LC–MS/MS analysis

To detect the level of cholesterol in hippocampus, we separated the hippocampus for liquid chromatography-tandem mass spectrometry (LC-MS/MS) analysis. Cholesterol was extracted from measured amounts of material (at least 50 mg per sample), which means a sample included four hippocampi from 2 to 4 mice randomly from same group. Cholesterol extracts were prepared by chloroform–methanol extraction, added in proper internal standards, and analyzed by 1290/6490 Triple Quadrupole LC/MS system (Agilent Technologies). Cholesterol was separated with normal-phase HPLC using a Waters Cortecs column (2.7 μm 2.1 × 100 mm Column) under the following conditions: mobile phase A (methanoic acid: water, 0.1:99.9, v/v) and mobile phase B (methanoic acid: methanol, 0.1:99.9, v/v); 95% A for 2 min, linear gradient to 30% A over 18 min and held for 3 min, and linear gradient to 95% A over 2 min and held for 6 min. Quantification of cholesterol was via multiple reaction monitoring. We measured the cholesterol in hippocampus by LC–MS/MS analysis, and the amount of cholesterol in every sample was normalized with their weight (μg/mg of wet tissue).

### Morris water maze

The MWM test to evaluate spatial learning consists of two consecutive stages: acquisition phase (AP) and space exploring test (SET) stage. Each mouse was given 4 trials per day for 5 days consecutive training with at least an hour intertrial interval. Each of the four starting positions was used randomly in daily trainings. In each trial, mice had 60 s to find platform in target quadrant. Trial was ended when mice arrived platform at least 3 s or time go to 60 s. When mice failed to find platform, they were guided to platform by experimenter and stayed about 20 s. Finally, space exploring test using trial as described previously but without the platform, and was 24 h later to the last training day. Tests were analyzed by Ethovision computerized tracking system (Noldus, Wageningen, Netherlands).

### Statistical analysis

All data were presented as mean ± SEM. All data subjected to statistical analysis in GraphPad Prism 9 (GraphPad Software, La Jolla, CA). Statistical significance between 3 or 4 groups (except escape latency in MWM test) were detected by one-way ANOVA with Tukey’s post hoc test. Statistical significance between two groups was analyzed by unpaired two-tailed Student’s t-test. Three-way repeated measures ANOVA was used to analyze the results of escape latency in MWM test, and post-hoc comparison was performed by Fisher’s least significant difference test. Differences with *P* < 0.05 were considered to be significant, and significance expressed as follows: *P < 0.05, **P < 0.01, ***P < 0.001.

## Results

### APP overexpression and /or Aβ overload leads to significant decrease of hippocampal cholesterol level in 5xFAD mice

**Fig. 1:**
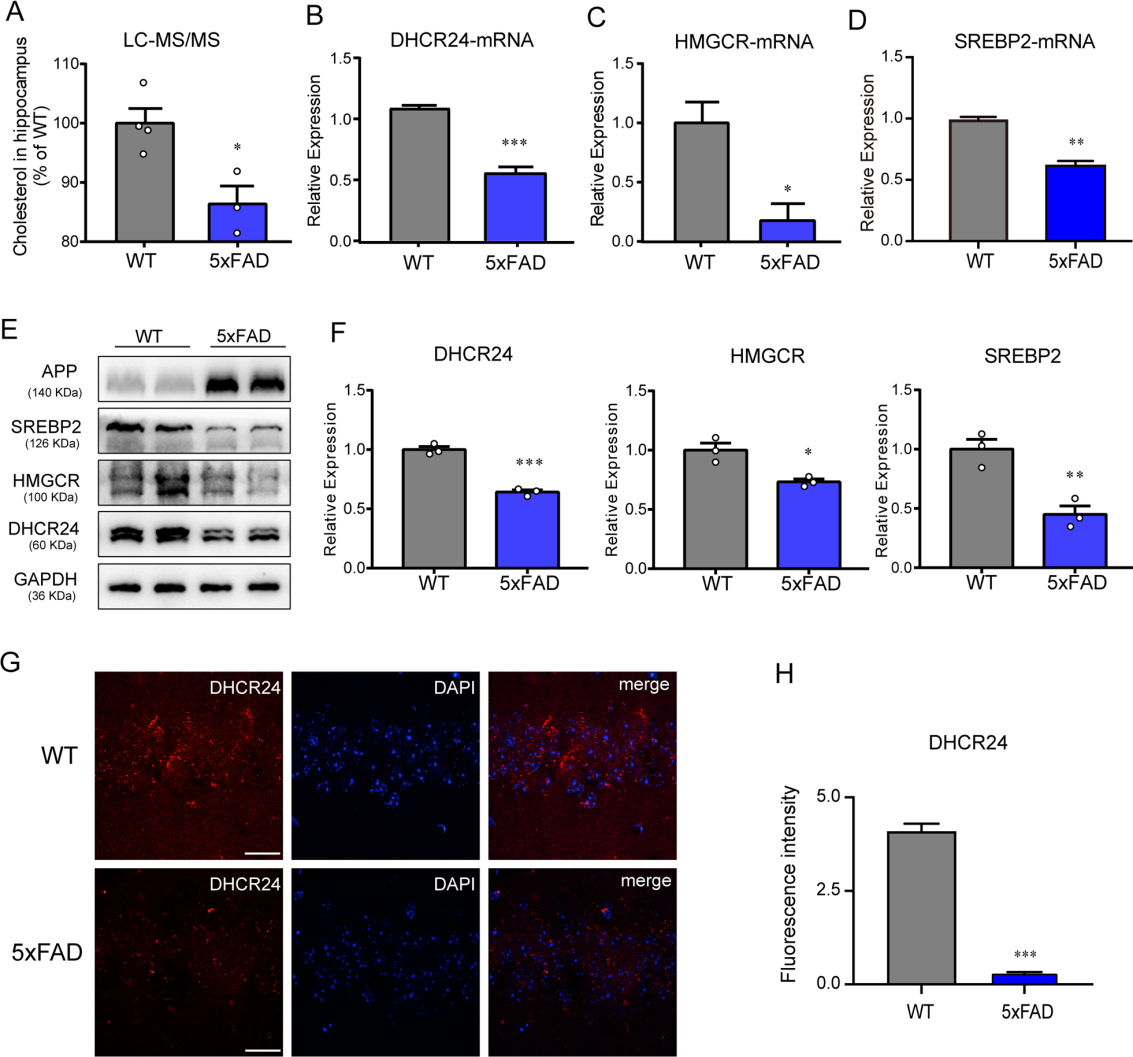
The significant decrease of hippocampal cholesterol level in 5xFAD mice. **A** Quantification of cholesterol level in hippocampus of WT and 5xFAD mice (3 month) by LC–MS/MS analysis. n = 3–4 sample/group, every sample was included 2–3 mice randomly. **B**–**D** The fold change expression of mRNA of DHCR24, HMGCR and SREBP2 in hippocampus of WT and 5xFAD mice (3 month) by RT-PCR. **E** The immunoblotting bands of DHCR24, HMGCR, SREBP2 in hippocampus of 3-month-old WT mice and 5xFAD mice. **F** Analysis of western blot with mean gray value which all were quantification on the ratio of target proteins against GAPDH. **G** The fluorescence images of DHCR24 (red) in hippocampus of 3-month-old WT and 5xFAD mice (scale bar, 20 μm). **H** Mean fluorescence intensity of DHCR24. n = 3 mice per group in [B-H]. Data expressed as mean ± SEM, statistical analysis was performed using unpaired two-tailed Student’s t-test. *P < 0.05; **P < 0.01; ***P < 0.001; compared with aged-matched WT group

Noticeably, in FAD mice brain, the rapid increase of Aβ could induce the inhibition of cholesterol synthetic genes, leading to cholesterol loss [6, 40, 71, 79]. And what’s more, previous studies have showed that APP and Aβ40 or Aβ42 inhibited cholesterol synthesis and decreased cellular cholesterol level in neuron or glia [6, 40, 57, 79]. To better understand the hippocampal cholesterol metabolism state of the early symptomatic 5xFAD mice, we investigated whole-hippocampus cholesterol metabolism on 3-month-old WT and 5xFAD mice. Moreover, western blot and RT-PCR showed that the mRNA and protein level of main genes involving in cholesterol synthesis, including sterol regulatory element binding protein 2 (SREBP2), 3-hydroxy-3-methylglutaryl-CoA reductase (HMGCR), and DHCR24, was markedly down-regulated in hippocampus of 5xFAD mice compared to WT mice, indicating inhibition of brain cholesterol synthesis (Fig. 1B–F). Furthermore, by a confocal laser scanning microscope, we observed that fluorescence intensity of the DHCR24 expression in hippocampus of 5xFAD mice was weaker than that of the age-matched WT mice, suggesting the decrease of hippocampal cholesterol synthesis (Fig. 1G). In contrast, immunofluorescence intensity analysis revealed that the level of hippocampal DHCR24 expression were lowered in the 5xFAD mice, indicating the decrease of hippocampal cholesterol biosynthesis level (Fig. 1H). Besides, compared with the WT mice, LC-MS/MS analysis indicated the cholesterol level of hippocampus is lower in 5xFAD mice (Fig. 1A).

Overall, based on previous research and our present study, we found the expression of essential genes involved in cholesterol synthesis were downregulated, resulting in a significant decrease of cholesterol level in the hippocampus of 5xFAD mice. Thus, a remarkable decrease in brain cholesterol level was appeared in the transgenic AD models, which means that these FAD mice are proper tools to investigate abnormal cholesterol metabolism associated with AD.

### DHCR24 overexpression increases cholesterol level in the hippocampus of 5xFAD mice and reverses cognitive impairment

**Fig. 2:**
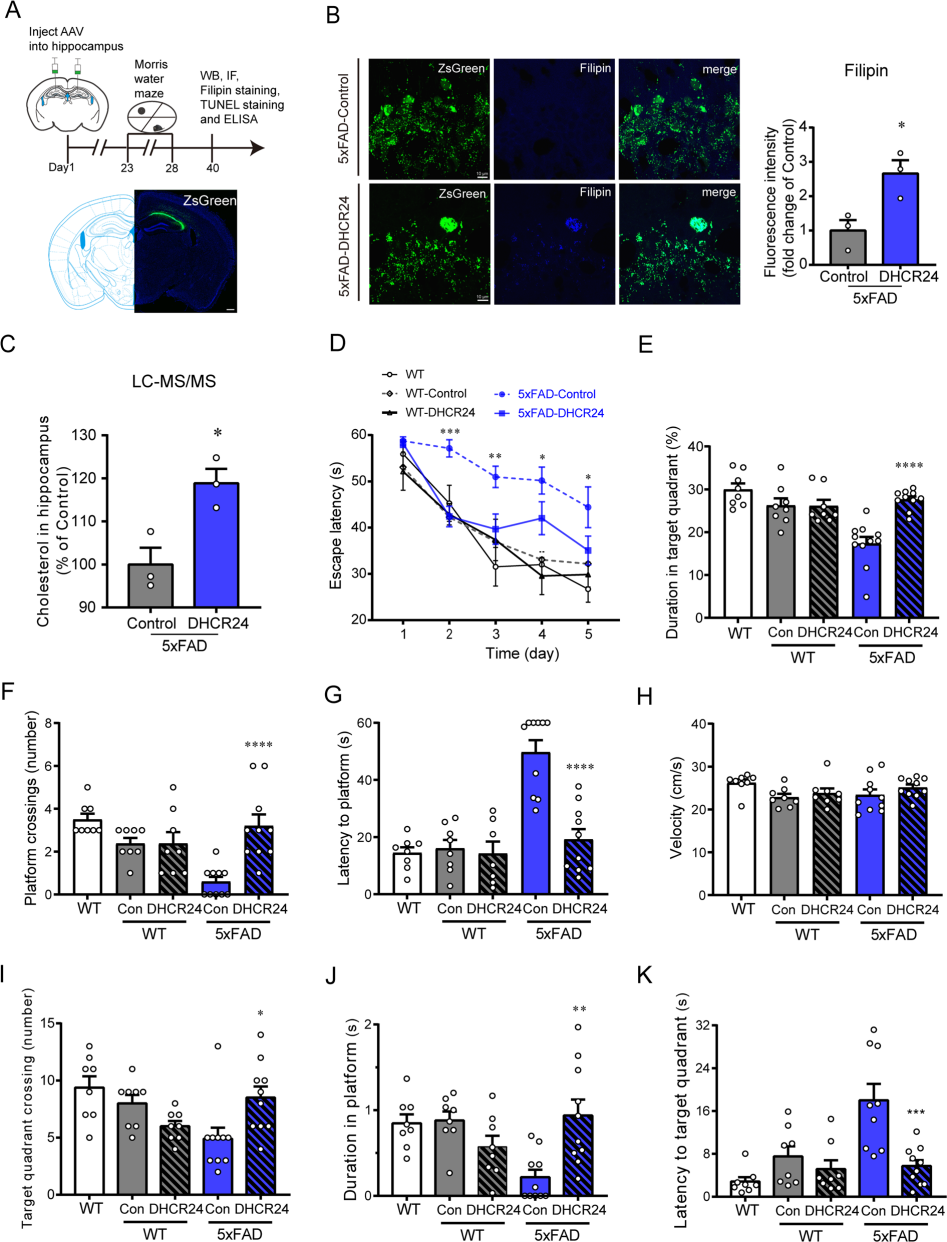
DHCR24 knock-in markedly improves cognitive ability of 5xFAD mice. **A** The timeline of the experiment and fluorescence images of ZsGreen (green) one month after AAV injection (scale bar, 200 μm). **B** Fluorescence images of Filipin staining (blue) in the CA1 region of hippocampus (scale bar, 10 μm) and the analysis results of mean fluorescence intensity which were normalized with 5xFAD-Control group. **C** Quantification of cholesterol level in hippocampus two groups by LC–MS/MS analysis which were normalized with 5xFAD-Control group. n = 3 sample/group, every sample was included 2–4 mice randomly. **D** Escape latency during the 5 days acquisition phase of Morris water maze (MWM) test. **E** The percentage of time spent in target quadrant in MWM test. **F** The number of platform crossings in MWM test. **G** The latency to platform in MWM test. **H** The swim speed of mice in MWM test. **I** The number of target quadrant crossings in MWM test. **J** The time spent in platform in MWM test. **K** The latency to the target quadrant in MWM test. n = 7–10 mice per group in [G], n = 8–10 mice per group in [**D**–**F** and** H**–**K**]. Data expressed as mean ± SEM, statistical analysis between the five groups was analyzed by one-way ANOVA with Tukey’s post hoc test, except for escape latency was analyzed by three-way repeated measures ANOVA with LSD post hoc test. *P < 0.05; **P < 0.01; ***P < 0.001; compared with aged-matched 5xFAD-Control group. The images of MWM trials were showed in Additional file 1: Fig. S1

To demonstrate the contribution of DHCR24 in improving the cognitive ability of AD, we employed delivery of AAV carrying DHCR24 gene into the hippocampus of 5xFAD mice at age of 3 months, and behavioral tests were performed at around 4-month-old (Fig. 2A). After DHCR24 transfection, the Filipin staining and LC-MS/MS analysis showed that the cholesterol level in the hippocampus of 5xFAD mice was up-regulated (Fig. 2B, C). To detect the effect of DHCR24 knock-in on cognition of 5xFAD mice, the Morris water maze (MWM) test was carried out [82]. The escape latency decreased significantly in 5xFAD-DHCR24 group after DHCR24 knock-in (Fig. 2D). The platform crossover number and the duration in target quadrant in 5xFAD-DHCR24 group were higher than that in the 5xFAD-control group (Fig. 2E, F). And the latency to platform and target quadrant decreased significantly after DHCR24 knock-in treatment in 5xFAD mice (Fig. 2G, K). The crossing number of target quadrant and duration in platform also significantly increased after DHCR24 overexpression in 5xFAD mice (Fig. 2I, J). In the trials, the swimming speed of mice in different groups was not significantly different (Fig. 2H). The results of MWM test suggested that DHCR24 knock-in effectively enhanced the cognitive ability of 5xFAD mice.

**Fig. 3:**
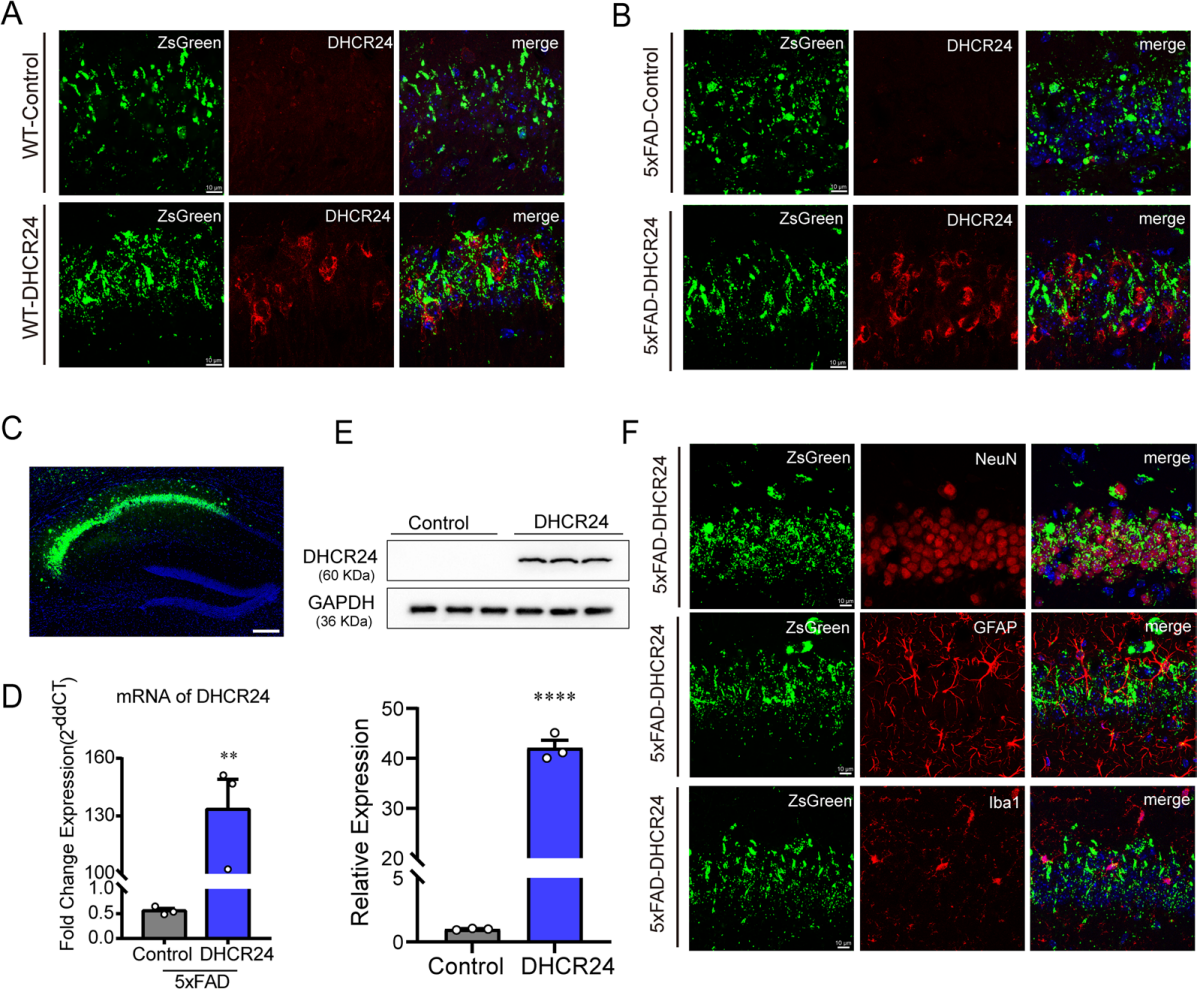
The over-expression of DHCR24 in the hippocampus of 5xFAD mice. **A**, **B** The fluorescence images of ZsGreen (green) and DHCR24 (red) in hippocampus (scale bar, 10 μm). **C** Fluorescence image of ZsGreen (green) in hippocampus (scale bar, 200 μm). **D** The fold change expression of mRNA of DHCR24 in hippocampus by RT-PCR. **E** The immunoblotting bands of DHCR24 protein and the analysis results of western blot with mean gray value which were quantification on the ratio of target proteins against GAPDH. n = 3 mice per group. **F** Co-staining of ZsGreen (green) with NeuN (red) and GFAP (red) or Iba1 (red) in hippocampus (scale bar, 10 μm). Data expressed as mean ± SEM, statistical analysis between the two groups was analyzed by unpaired two-tailed Student’s t-test. *P < 0.05; **P < 0.01; ***P < 0.001; compared with aged-matched 5xFAD-Control group

About one month after AAV injection, we detected ZsGreen fluorescence to verify successful injection, and found that ZsGreen is specifically expressed in the hippocampal CA1, CA2 and CA3 regions (Fig. 3C). After DHCR24 transfection, compared with 5xFAD-control mice, we found that DHCR24 is over-expressed in the neurons and astrocytes, as well as rarely in microglial cells, in the hippocampus of 5xFAD-DHCR24 group, indicating DHCR24 is successfully transfected (Fig. 3B, F). Similarly, we found that DHCR24 is overexpressed in the hippocampal CA1, CA2, and CA3 regions of WT-DHCR24 group (Fig. 3A). RT-PCR and western blot analysis revealed the mRNA and protein level of DHCR24 expression in the hippocampus is increased by roughly 200 folds and by roughly 40 folds, respectively after DHCR24 knock-in treatment in 5xFAD mice (Fig. 3C–E). Above data indicated DHCR24 overexpression in neurons and astrocytes could lead to the increase of astrocytic and neuronal cholesterol synthesis, resulting in elevated level of cellular cholesterol in the hippocampus. Overall, our outcomes suggested that DHCR24 knock-in could reverse the cognitive impairment of 5xFAD mice.

### DHCR24 knock-in reduces Aβ deposition in hippocampus of 5xFAD mice

**Fig. 4:**
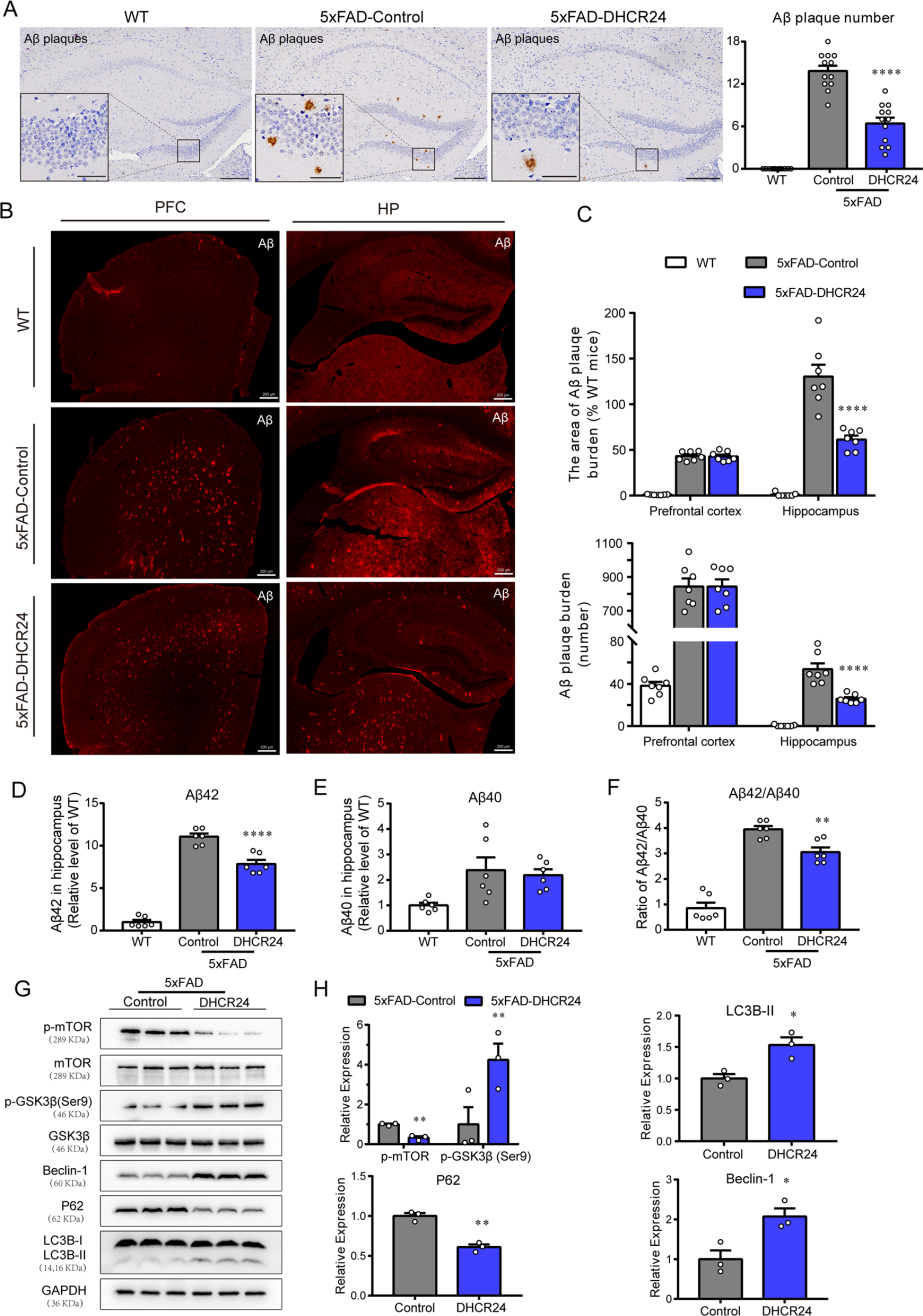
DHCR24 knock-in alleviates Aβ pathology and activates autophagy flux in hippocampus of 5xFAD mice. **A** The immunohistochemistry images of Aβ plagues using the antibody MOAB-2 in the hippocampus (Scale bars, 200 μm). Insets show a higher magnification of Aβ plagues. Inset scale bar, 50 μm. The right is analysis of number of Aβ plagues in hippocampus. n = 12 slices from 4 mice per group. **B** Fluorescence images of Aβ (red) in prefrontal cortex and whole hippocampus (scale bar, 200 μm). **C** The top half is the analysis of the area of Aβ plagues, and the bottom half is the analysis of number of Aβ plagues in prefrontal cortex and hippocampus. n = 7 slices from 4 mice per group. **D**, **E** The relative level of Aβ42 and Aβ40 in hippocampus by ELISA which normalized with WT mice. n = 6 mice per group. **F** The ratio of Aβ42/Aβ40 in hippocampus which normalized with WT mice. **G** The immunoblotting bands of p-mTOR, p-GSK3β (ser9), Beclin-1, P62 and LC3B in the hippocampus of 5xFAD-Control group and 5xFAD-DHCR24 group. **H** Analysis of western blot with mean gray value which all were quantification on the ratio of target proteins against GAPDH except p-mTOR and p-GSK3β (ser9) was against total mTOR and GSK3β, n = 3 mice per group. Data expressed as mean ± SEM, statistical analysis between two groups was analyzed by unpaired two-tailed Student’s t-test, between three groups was analyzed by one-way ANOVA with Tukey’s post hoc test. *P < 0.05; **P < 0.01; ***P < 0.001; compared with aged-matched 5xFAD-Control group

Altered neuronal membrane cholesterol and/or subcellular level have been implicated in aberrant production and aggregation of Aβ peptides [1, 6, 10, 15, 26, 47]. Interestingly, compared with 5xFAD-control group, we found DHCR24 overexpression decreased the Aβ deposits and plaques in the hippocampal regions of 5xFAD mice (Fig. 4A–C). Further, the level of soluble Aβ42 and ratio of soluble Aβ42/Aβ40 is significantly lowered, and there’s also a trend downward in the level of soluble Aβ40 in hippocampus of 5xFAD mice after DHCR24 knock-in (Fig. 4D–F). To sum up, our outcomes suggested that DHCR24 knock-in markedly reduced the content of soluble Aβ42 and Aβ deposition. Of note, in neuronal and animal models, previous studies reported that deficiency of cellular cholesterol increased the generation of Aβ40 and Aβ42 [1, 6, 15]. Conversely, increasing cellular cholesterol level markedly reduced the production of Aβ40 and Aβ42 [1, 6, 15]. Very importantly, genetic evidences also support that neuronal cholesterol deficiency is associated with Aβ production and accumulation, which is consistent with our present findings [6, 10, 21, 47, 71, 74]. Taking together, our findings revealed that increasing cellular cholesterol level by DHCR24 knock-in could prevent or reverse Aβ deposition in 5xFAD mice.

### DHCR24 knock-in reverses the inhibition of autophagy in 5xFAD mice

Autophagy is an important pathway for the clearance of aggregation-prone proteins including Aβ involved in AD [39, 44, 59, 78]. In the initial step of autophagy, it involves numerous proteins such as the autophagy-related (ATG) proteins, which including Beclin-1 and microtubule associated protein 1 light chain 3 Alpha (LC3). Interestingly, Beclin-1, which controlling an early step in the forms of autophagosome, was found to be downregulated in the brains of AD patients [39]. In our experiment, compared with the 5xFAD-control group, the expression of Beclin-1 and the LC3BII/LC3BI ratio increased in hippocampus of 5xFAD-DHCR24 group, implicating autophagy is activated (Fig. 4G–H). Furthermore, we found DHCR24 knock-in lowered the expression of Sequestosome1 (p62/SQSTM1), suggesting that degraded targets are recruited to autophagosomes via p62, a marker of autophagic activity (Fig. 4G, H).

In addition, the kinase mammalian target of rapamycin (mTOR) is an important regulator of the autophagy [6, 44]. And mTOR activity is modulated by upstream pathways, such as glycogen synthase kinase-3beta (GSK-3β), which are regulated by DHCR24 [6, 78]. Here, we showed that 5xFAD mice had lower expression level of phosphorylated mTOR (p-mTOR) and higher expression level of phosphorylated GSK3β at Ser9 site after DHCR24 knock-in, suggesting DHCR24 overexpression induced the inhibition of mTOR signaling by GSK3β inactivation (Fig. 4G, H). Similarly, we also found DHCR24 overexpression activated autophagy by the cholesterol-mediated lipid-dependent mTOR signaling in cultured cells [6]. Moreover, it found that mTOR signaling regulates autophagy, and be inhibited in cortex or hippocampus of AD model mice [44, 78]. And autophagy disorder is a well consensus of participating mechanism in AD neuropathology, which is associated with memory impairment [44, 59, 78]. Therefore, our outcomes demonstrated DHCR24 knock-in could prevent or reverse the inhibition of AD-related autophagy in 5xFAD mice, at least partly via modulating cholesterol-mediated GSK3β/mTOR pathway.

### DHCR24 overexpression reverses hippocampal synaptic injuries

**Fig. 5:**
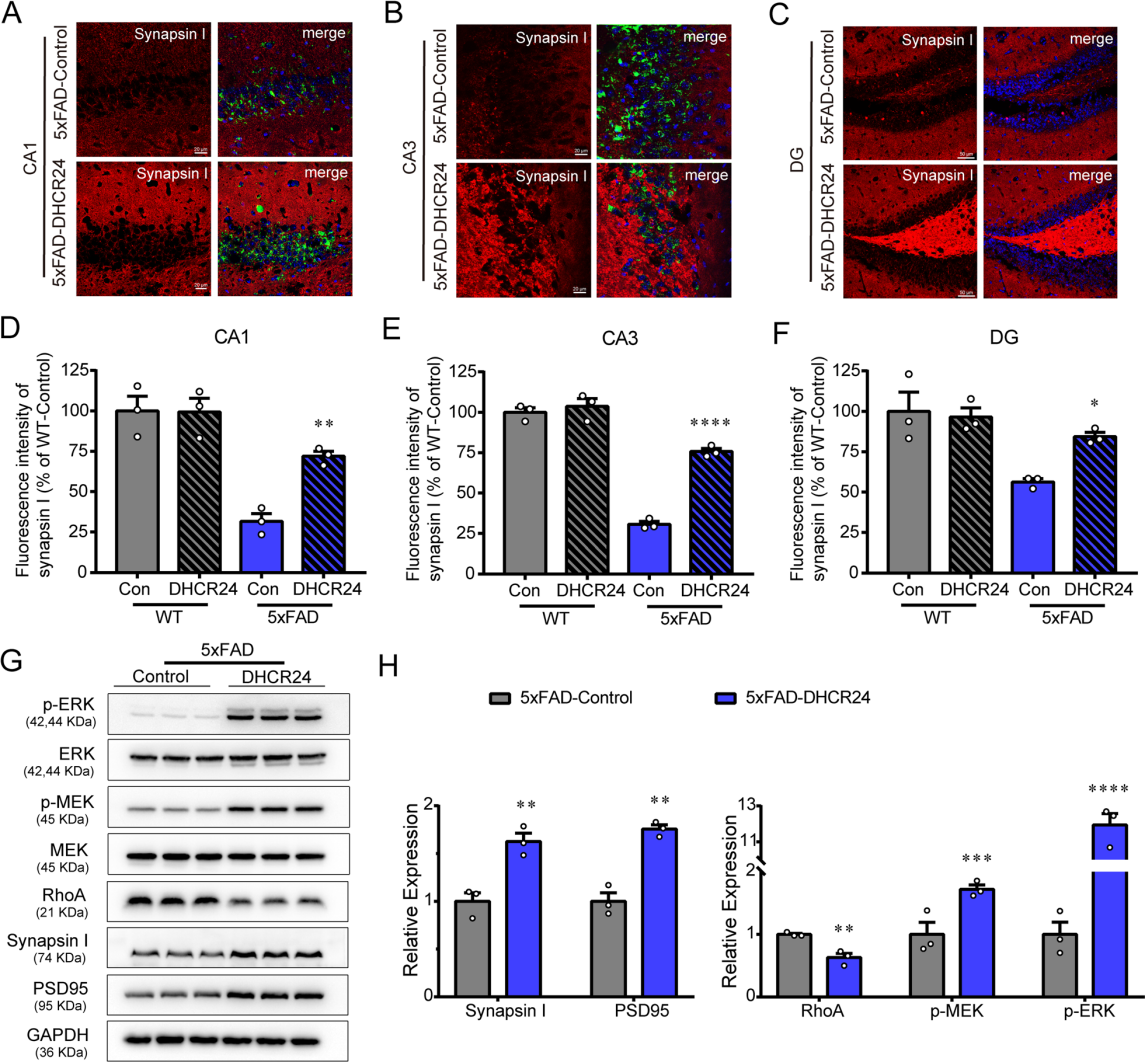
DHCR24 knock-in improves synaptic function in 5xFAD mice. **A**, **B** Fluorescence images of Synapsin I in the CA1 and CA3 region of hippocampus (scale bar, 20 μm). **C** Fluorescence images of Synapsin I in the DG region of hippocampus (scale bar, 50 μm). **D**–**F** Mean fluorescence intensity of Synapsin I in the CA1, CA3, and DG region. n = 3 mice/group. **G** The immunoblotting bands of PSD95, Synapsin I, RhoA, p-MEK and p-ERK in the hippocampus of 5xFAD-Control group and 5xFAD-DHCR24 group. **H** Analysis of western blot with mean gray value which all were quantification on the ratio of target proteins against GAPDH except p-MEK and p-ERK were the ratio against total MEK and ERK. n = 3 mice/group. Data expressed as mean ± SEM, statistical analysis between the two groups was analyzed by unpaired two-tailed Student’s t-test, between the four groups was analyzed by one-way ANOVA with Tukey’s post hoc test. *P < 0.05; **P < 0.01; ***P < 0.001; compared with aged-matched 5xFAD-Control group. The images of Synapsin I in CA1, CA3 and DG regions of WT-Control group and WT-DHCR24 group were showed in Additional file 2: Fig. S2

Of note, previous studies confirmed that the formation of numerous and efficient synapses of neurons need glia-supplied cholesterol, which influencing basic synapse function, plasticity and behavior [50, 81]. Conversely, cellular cholesterol deficiency by genetic defects in cholesterol trafficking can impair synaptic growth and plasticity, and synaptic function, such as transmission, and then contribute to cognitive deficits [10, 60, 81]. Here, the presynaptic marker synapsin-1 was performed in the hippocampal region by immunofluorescent (IF) staining (Fig. 5A–C). We observed that 5xFAD mice showed increased synapsin-1 immunoreactivity in the CA1, CA3, and dentate gyrus (DG) regions of hippocampus after DHCR24 knock-in (Fig. 5A–C). And the IF analysis demonstrated that synapsin-1 in 5xFAD mice exhibited a 2.3-fold increase in the CA1 region, a 2.5-fold increase in the CA3 region and a 1.5-fold increase in the DG region after DHCR24 overexpression (Fig. 5D–F). However, there was no significant difference in synapsin-1 immunoreactivity in WT-control group and WT-DHCR24 group (Additional file 2: Fig. S2). Additionally, western blot analysis revealed that a dramatic increase in both the postsynaptic density 95 (PSD95) and synapsin-1 protein expression levels in hippocampus of 5xFAD mice after DHCR24 knock-in (Fig. 5G, H). Overall, our results revealed DHCR24 knock-in significantly reversed the decrease of PSD95 and synapsin-1 protein expression in hippocampus of 5xFAD mice, suggesting the increase of synapse number in hippocampus. Notably, DHCR24 mutation or knockout leads to the loss of membrane cholesterol and disorder of lipid raft in the brain of patients and mice, resulting in synaptic abnormality and cognitive deficits [2, 6, 33, 65, 75, 88]. In contrast, DHCR24 knock-in increased the number of synapses in mouse hippocampal neurons, promoting synapse formation [49]. Therefore, the increase of hippocampal neuronal cholesterol level by DHCR24 knock-in could promote synapse formation and maturation, and then improve the synapse function, which may be a mechanism by which cholesterol in plasma membrane affects synaptic transmission.

In addition, in the present study, DHCR24 knock-in increased the level of extracellular signal-related kinases 1 and 2 (ERK1/2) phosphorylation in the hippocampus of 5xFAD-DHCR24 group compared to 5xFAD-control group, suggesting ERK1/2 signaling was activated (Fig. 5G, H). ERK1/2 pathway regulated experience-dependent gene transcription, which has been proved to be essential to experience-related synaptic plasticity and formation of long-term memory [70]. Meanwhile, a significant decrease in RhoA levels in the Rho GTPase pathway was observed after DHCR24 knock-in treatment in 5xFAD mice (Fig. 5G, H). Collectively, our outcomes supported that DHCR24 knock-in regulated cholesterol-dependent lipid raft-related signals, including ERK1/2 and Rho-GTPase pathways in the hippocampus, which are involved in modulating synaptic plasticity and reconsolidation of memory in AD [3, 43, 70]. Hence, our outcomes supported that memory-enhancing activity could be associated with the enhancement of hippocampal synaptic function through DHCR24-mediated increase of cellular cholesterol level.

### DHCR24 overexpression prevents hippocampal cellular apoptosis

**Fig. 6:**
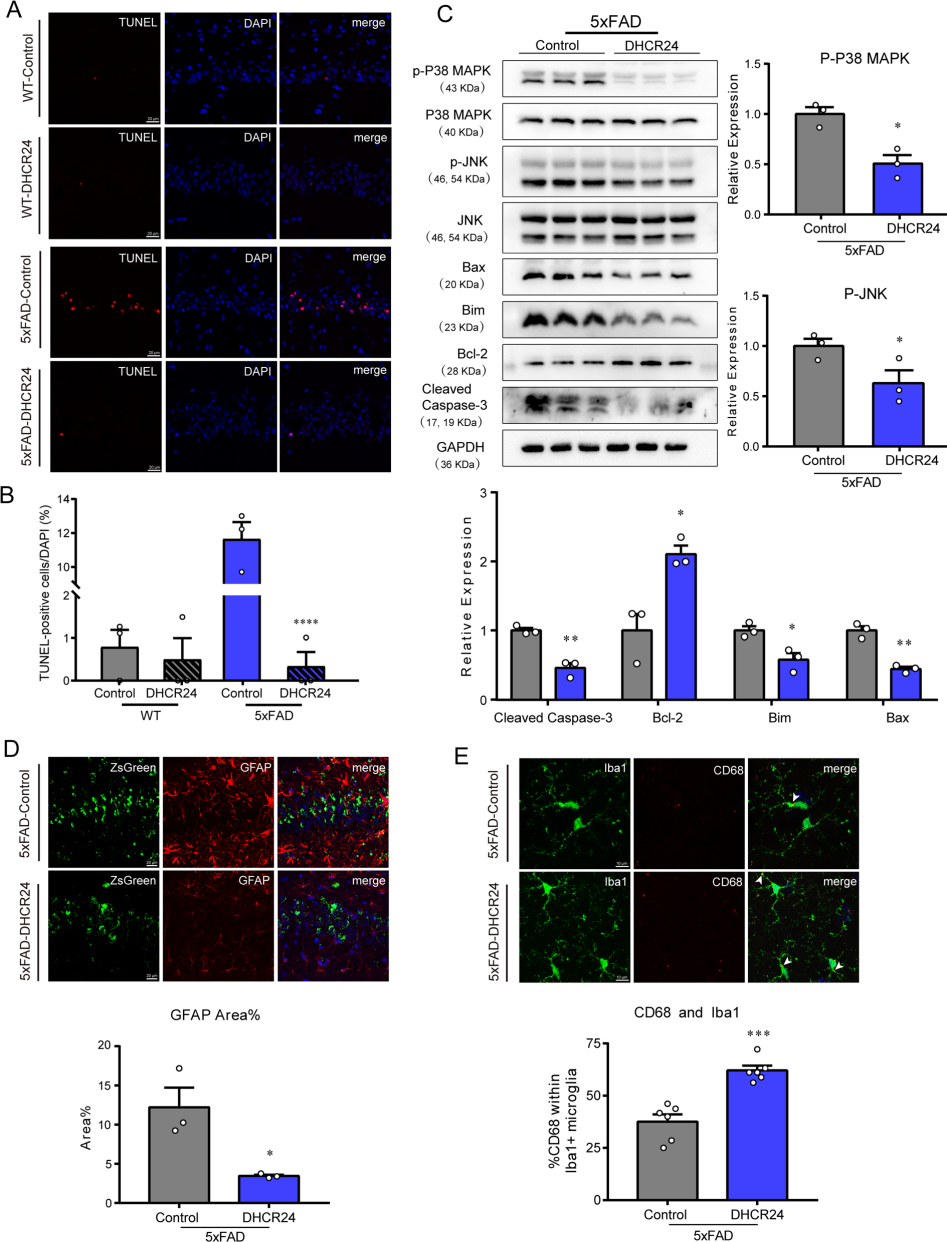
DHCR24 knock-in inhibits apoptosis or reactive astrocytosis and promotes microglial phagocytosis in 5xFAD mice. **A** Fluorescence images of TdT-mediated dUTP Nick-End Labeling (TUNEL) staining (red) in the CA1 region of hippocampus (scale bar, 20 μm). **B** The ratio of TUNEL-positive cells against DAPI. **C** Representative immunoblotting bands of Cleaved caspase3, Bcl-2, Bim, Bax, p-JNK and p-P38 MAPK and analysis of western blot with mean gray value which all were quantification on the ratio of target proteins against GAPDH except p-JNK and p-P38 MAPK were against total JNK and P38 MAPK. **D** Co-staining of ZsGreen (green) with GFAP (red) in hippocampus and analyzing with the percentage of fluorescence area of GFAP (scale bar, 20 μm). **E** Co-staining of Iba1 (green) with CD68 (red) in hippocampus and analyzing with the percentage of CD68 + positive within Iba1 + microglia (scale bar, 10 μm). The arrows indicate CD68 + and Iba1 + microglia. n = 3 mice/group in [A-D], n = 6 brain slices from 3 mice/group in [E]. Data expressed as mean ± SEM, statistical analysis between the two groups was analyzed by unpaired two-tailed Student’s t-test, between the four groups was analyzed by one-way ANOVA with Tukey’s post hoc test. *P < 0.05; **P < 0.01; ***P < 0.001; compared with aged-matched 5xFAD-Control group

Previous research revealed that DHCR24 can regulate cell survival and death, which is associated with the change of cellular cholesterol level [6, 67]. Here, we did not find any noticeable TUNEL-positive cells in the hippocampus of the WT-control or WT-DHCR24 group (Fig. 6A, B). In contrast, TUNEL-positive cells were more observed in the CA1 pyramidal cell layers in the 5xFAD-control group, suggesting abnormal apoptosis in hippocampus (Fig. 6A, B). Importantly, TUNEL positive cells were decreased in hippocampus of 5xFAD mice after DHCR24 knock-in treatment (Fig. 6A, B). Accordingly, these results revealed that DHCR24 knock-in prevented or reversed apoptotic activity in the hippocampus of 5xFAD mice.

Intriguingly, the expression of B-cell lymphoma-2 (Bcl-2) family members, such as Bcl-2, Bcl-2-associated x protein (Bax), and building information modelling (Bim), was altered in AD brain, which controls cell death and survival processes by regulating mitochondrial outer membrane permeabilization [13, 68]. The mitogen-activated protein kinases (MAPK) family members, including c-Jun N-terminal kinase (JNK) and p38 kinase (P38), are known to regulate the activity of Bcl-2 family proteins [24, 68]. In our study, our results showed that the 5xFAD-DHCR24 group had lower levels of phosphorylated P38 (p-P38) and phosphorylated JNK (p-JNK), as well as higher level of Bcl-2 expression and lower levels of Bax and Bim expression (Fig. 6C). And the level of cleaved caspase-3 was markedly lowered in 5xFAD mice after DHCR24 knock-in (Fig. 6C). Furthermore, studies have shown that overexpression of DHCR24 protected cells from apoptosis by Aβ-mediated toxicity, which is consistent with our present outcomes [6, 25, 37, 41, 67]. Thus, our data again confirmed that the anti-apoptotic effect of DHCR24 knock-in could be via P38 and JNK pathways which are regulated by cholesterol-dependent membrane lipid-rafts in the brain of 5xFAD mice.

### DHCR24 overexpression inhibits reactive astrocytosis and promotes microglial phagocytosis

Previous research revealed that the Aβ secreted from APP overexpressing transgenic mouse models could stimulate reactive astrocytosis, which contributes to progression of AD [34, 54, 58]. Here, the immunofluorescence staining of GFAP showed that DHCR24 knock-in inhibited reactive astrocytosis (Fig. 6D), which may be related to the reduction of Aβ deposition. As for microglia, the phagocytic function was essential for the clearance of Aβ plaques, and research found that phagocytic activity of microglia to the extracellular matrix could promote synapse plasticity in brain [16, 34, 54]. In present study, our results showed that the 5xFAD mice had higher rate of CD68 positive within Iba1 positive microglial cells after DHCR24 knock-in (Fig. 6E). As CD68 is a marker of active microglial phagocytosis [51, 69], above results suggested DHCR24 knock-in promoted microglial phagocytosis in 5xFAD mice (Fig. 6E). Thus, our data indicated that DHCR24 knock-in could inhibit reactive astrocytosis by increasing microglial-mediated clearance of Aβ, and pro-phagocytic effects of DHCR24 could be the potential mechanism by which DHCR24 knock-in alleviates AD-related pathology and cognitive impairment.

## Discussion

In the present work, we found that there is a significant inhibition of hippocampal cholesterol biosynthesis, coupled with significantly decreased hippocampal cholesterol level in 5xFAD mice compared to age-matched WT mice (Fig. 1A–H). Moreover, the findings of our present study are consistent with previous research on FAD mice [6, 40, 57, 79]. In fact, APP overexpressing transgenic mouse models had lower level of cellular cholesterol in brain, and APP knockout mice had higher level of brain cellular cholesterol, which means that APP overexpression and/or Aβ overload leads to brain cholesterol loss [40, 79]. Consequently, our findings suggested that the transgenic FAD mice, including 5xFAD mice, could be used as model to study brain cholesterol deficiency associated with AD. In order to test the AD-cholesterol hypothesis that brain cellular cholesterol deficiency triggers the onset and progression of AD, we conducted a new cholesterol-based DHCR24 gene therapy in 5xFAD mice (Fig. 2A). Surprisingly, by employing delivery of AAV9 carrying DHCR24 gene into the hippocampus of 5xFAD mice, we found that DHCR24 knock-in significantly reversed the cognitive impairment (Fig. 2D–K). Thus, our outcome firstly demonstrated that the potential value of DHCR24 knock-in as a promising treatment for AD.

**Fig. 7:**
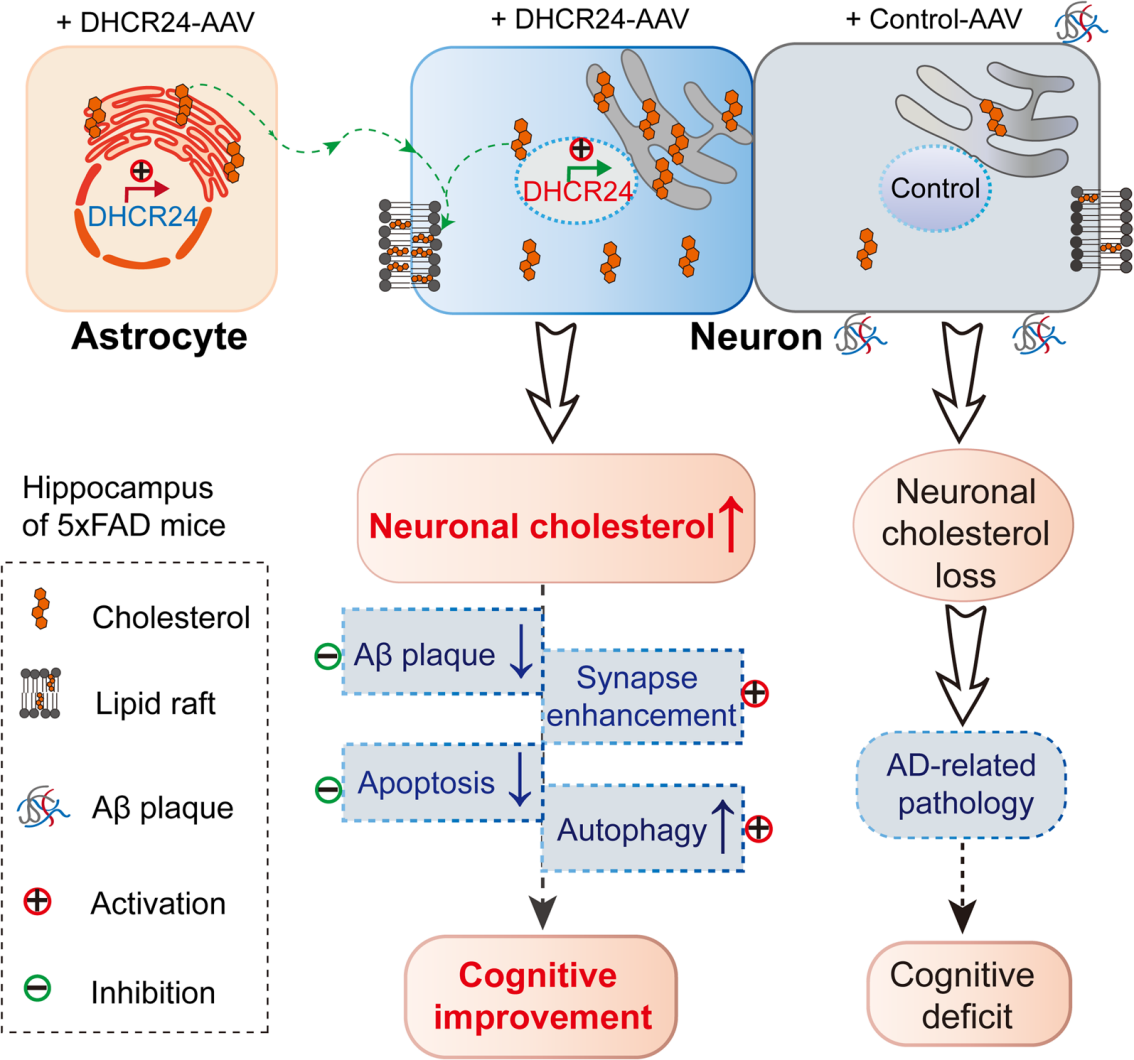
Schematic summary of DHCR24 knock-in reverses AD-related pathology and enhances cognitive ability of 5xFAD mice. To demonstrate the contribution of DHCR24 in improving the cognitive ability of AD, we employed delivery of adeno-associated virus (AAV) carrying DHCR24 gene into the hippocampus of 5xFAD mice. After DHCR24 transfection, DHCR24 is universally co-expressed in neurons and astrocytes, resulting in the increase of neuronal cholesterol level. By enhancing neuronal cholesterol level, DHCR24 knock-in successfully prevented or reversed AD-related pathology, including amyloid-β deposition, synaptic injuries, inhibition of autophagy, and apoptosis. Finally, DHCR24 knock-in obviously improved the cognitive ability of 5xFAD mice

Interestingly, we observed that DHCR24 is universally overexpressed in the hippocampal neurons and astrocytes of 5xFAD mice, coupled with the increase of cholesterol level in the hippocampus, indicating the increase of neuronal and astrocytic cholesterol synthesis in the hippocampus (Fig. 3B, F). In adult brain, the cholesterol-related activities of neurons are mainly supported by astrocyte-derived cholesterol, with a little part supported by cholesterol synthesized themselves [10, 21, 28, 62]. Accordingly, enhancement of DHCR24 function in the hippocampal neurons and astrocytes could induce an increase of cholesterol synthesis and supplementation, which could rectify the deficiency of neuronal cholesterol in 5xFAD mice brain (Fig. 7). Collectively, DHCR24 knock-in in astrocytes and neurons could increase cholesterol biosynthesis and rectify the situation of neuronal cholesterol deficiency, and then impact aspects of neuronal dysfunction in 5xFAD mice model (Fig. 7).

Very interestingly, in present study, we showed that DHCR24 knock-in could obviously prevent or reverse AD-related pathology, including amyloid-β deposition, synaptic injuries, inhibition of autophagy, and apoptosis, leading to a significant improvement in cognitive ability of 5xFAD model (Figs. 2, 3, 4, 5, 6, 7). Similarly, in vitro cultured cell models, previous studies have demonstrated that DHCR24 obviously could reverse AD-related pathological impairments by the increase of neuronal cellular cholesterol content [6, 7, 15, 36, 43, 50, 63]. Furthermore, within the brain, the majority of cholesterol is exited in cytoplasm, organelles and myelin sheaths [18, 61]. Neuronal morphology and function, as well as synaptic transmission, may require large amounts of cholesterol to maintain, as cholesterol was a vital membrane component and a regulator for signaling molecules [18, 81]. What’s more, increasing evidences strongly supported that the deficiency in brain cellular cholesterol could contribute to AD-related pathology [2–4, 6, 7, 15, 21, 25, 26, 36, 37, 41, 43, 47, 50, 60, 63, 65, 80, 81, 88]. Therefore, our outcomes strongly indicated that DHCR24-mediated gene therapy could be a promising approach against AD via increasing neuronal cholesterol level.

The cholesterol in brain is about 23% of all in body [18, 28]. Mechanistically, brain cellular cholesterol is a useful molecule, which serves as initial membrane component, as a ligand or cofactor for proteins and as precursor for neurosteroid [6, 18, 30, 46, 53, 72, 81, 84]. Firstly, as an essential lipid molecule, cholesterol plays a critical role in the homeostasis of membrane structure and function. Moreover, cholesterol loss could lead to the disorders of lipid rafts and affect membrane-mediated cell activities and signaling transduction, such as membrane-related activity, and signaling including PI3K/Akt, GSK3, mTOR, MAPK/ERK, P38 MAPK, JNK, which has been linked to AD pathogenesis [6, 46, 53, 84]. Secondly, as a ligand or cofactor for proteins, the direct impact on free (unesterified) cholesterol on the structure and function of membrane proteins or intracellular proteins is recognized [20, 30, 72]. Moreover, it is found more than 250 candidate cholesterol-binding proteins in mammalian cells, including receptors, channels, and enzymes [30]. And free cholesterol may bind to proteins as a covalent or an allosteric modulator of protein function, such as G-protein coupled receptors (GPCR) and trafficking proteins and alters localization, transport or sorting of proteins between different organelles or membrane domains [20, 30, 72]. Thus, free cholesterol itself could directly regulate the structure or function of proteins by the specific interactions with proteins in mammalian cells, which could contribute to AD pathogenesis [6, 20, 30, 72]. Thirdly, Cholesterol in the brain is also the precursor for neurosteroid biosynthesis [18, 28, 62, 81]. Previous data demonstrated neurosteroid hormone played a wide variety of essential protective and regulatory roles in the brain [11, 38, 85, 86]. The decrease or deficiency of neurosteroids in the brain is related to aging and neurodegenerative diseases, such as AD [38, 86]. Hence, increasing evidences strongly support that brain cholesterol plays a determined influence in brain development, homeostasis, and functional modulation.

Very importantly, our present study reflected on how DHCR24 knock-in markedly impacted critical aspects of AD pathology in 5xFAD model, which is consistent with previous studies in cell models, supporting brain cellular cholesterol is a key modulator of AD-related pathology [6, 7, 10, 14, 15, 21, 36, 43, 47, 60, 63, 76, 80]. Intriguingly, on one hand, in many AD models and patients, increasing data indicated that the genetic and non-genetic risk factors for AD could lead to brain cholesterol deficiency, which means a direct causative link between brain cholesterol loss and AD (FAD and SAD) [6, 10, 21, 26, 47, 71, 74]. On the other hand, genetic evidences and outcomes from cell models strongly support cellular cholesterol deficiency contributes to AD pathological impairments [6, 10, 21, 71, 74]. Therefore, compelling evidences support that brain cellular cholesterol loss could induce the initiation and progression of AD [6]. Notably, in spite of etiology and background heterogeneous in FAD and SAD, the common pathological and clinical features for FAD and SAD support that the underlying mechanisms of damage to neurons seem to be very similar [6, 10, 14, 36, 47, 60, 80]. Based on previous research and our present study, we concluded that neuronal cholesterol deficiency could play a critical role in modulating AD-related pathology, which is very likely to be a common node-point that triggers the initiation and progression of FAD and SAD. Therefore, our present study firstly demonstrated that brain cellular cholesterol deficiency could contribute to the pathogenesis of AD in 5xFAD model.

In summary, in present study, we showed cholesterol-based DHCR24 knock-in prevented or reversed AD-related pathology and effectively improved the cognitive impairment of 5xFAD mice by enhancement of neuronal cholesterol level. Consequently, encouraging results from our present study of DHCR24-targeted gene delivery in the brain could lead to the transition from pre-clinical to clinical trials. Besides, targeting the common node-point of brain cellular cholesterol deficiency, gene therapy might be a suitable treatment of AD compared to conventional therapy since it can be tailored to specifically alter AD-related pathological impairments, reverse disease phenotype and restore brain normal function. Thus, modulation of brain cellular cholesterol level by the target genes involving in cellular cholesterol synthesis or trafficking, such as HMGCR, DHCR24, APOE2, and LDLR, could be very valuable for preventing or reversing AD pathology [3, 6, 29, 33, 76, 87]. Accordingly, the rectification of cellular cholesterol deficiency/loss in AD brain could be a potential treatment to prevent or slow the neurodegeneration of AD.

## Supplementary Information

**Additional file1: Fig. S1**. Representative images of the Morris water maze trials of the mice of five groups

**Additional file 2: Fig. S2**. The effect of DHCR24 knock-in on Synapsin I in the hippocampus of WT mice. **A** Fluorescence images of Synapsin I in the CA1 region in WT-Control group and WT-DHCR24 group. **B** Mean fluorescence intensity of Synapsin I in the CA1 region. **C** The images of Synapsin I in the CA3 region in WT-Control group and WT-DHCR24 group. **D** Mean fluorescence intensity of Synapsin I in the CA3 region. **E** Fluorescence images of Synapsin I in the DG region. **F** Mean fluorescence intensity of Synapsin I in the DG region. n = 3 mice per group in [A-F]. Data expressed as mean ± SEM, statistical analysis between the two groups was analyzed by unpaired two-tailed Student’s t-test. *P < 0.05; **P < 0.01; ***P < 0.001; compared with aged-matched WT-Control group

## Data Availability

This study includes no data deposited in external repositories. Expanded View for this article is available online. DHCR24 gene source data, other data that support the findings of this study are available from the corresponding author upon reasonable request.

## Abbreviations

AAV: Adeno-associated virus
ABC: ATP binding cassette
Aβ: Amyloid-β
AD: Alzheimer’s disease
APOE4: Apolipoprotein E4
APP: Amyloid precursor protein
ATG: Autophagy-related protein
Bax: BCL-2-Associated X Protein
Bcl-2: B-cell lymphoma-2
Bim: Bcl-2 interacting mediator of cell death
DHCR24: 24-Dehydrocholesterol reductase
ERK1/2: Extracellular-regulated kinase 1/2
FAD: Familial Alzheimer’s disease
GPCR: G-protein coupled receptors
GSK-3β: Glycogen synthase kinase-3beta
HMGCR: 3-Hydroxy-3-methylglutaryl-CoA reductase
LC3: Microtubule associated protein 1 light chain 3 Alpha
LC-MS/MS: Liquid chromatography-tandem mass spectrometry
LDLR: Low-density lipoprotein receptor
IF: Immunofluorescent
JNK: C-Jun N-terminal kinase
MAPK: Mitogen-activated protein kinases
mTOR: Mammalian target of rapamycin
MWM: Morris water maze
NPC1/2: Niemann Pick type C1/2
p62/SQSTM1: Sequestosome1
p38 MAPK: P38 mitogen activated protein kinases
PSD95: Postsynaptic density 95
SREBP2: Sterol regulatory element binding protein 2
SAD: Sporadic Alzheimer’s disease
Tau: Microtubule-associated protein tau
TUNEL: DTdT-mediated dUTP Nick-End Labeling
WT: Wild-type
