# Supplementary figures and images for: DHCR24 reverses Alzheimer’s disease-related pathology and cognitive impairment via increasing hippocampal cholesterol levels in 5xFAD mice
Source: Acta Neuropathol Commun. 2023 Jun 21;11:102. doi: 10.1186/s40478-023-01593-y (PMC10286507; doi:10.1186/s40478-023-01593-y)

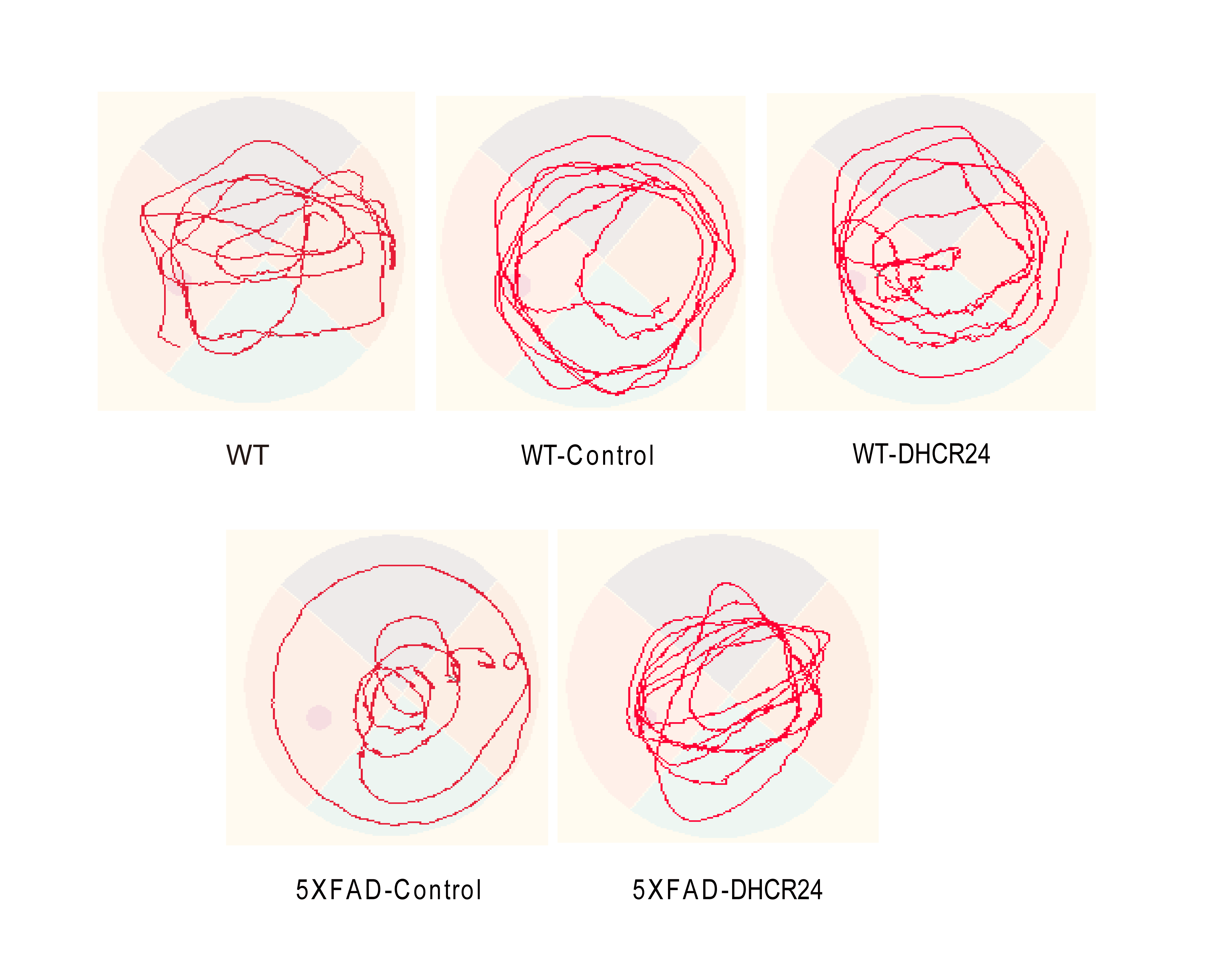

Supplement: Supplementary file 1 — Additional file1: Fig. S1. Representative images of the Morris water maze trials of the mice of five groups [file 40478_2023_1593_MOESM1_ESM.tif]

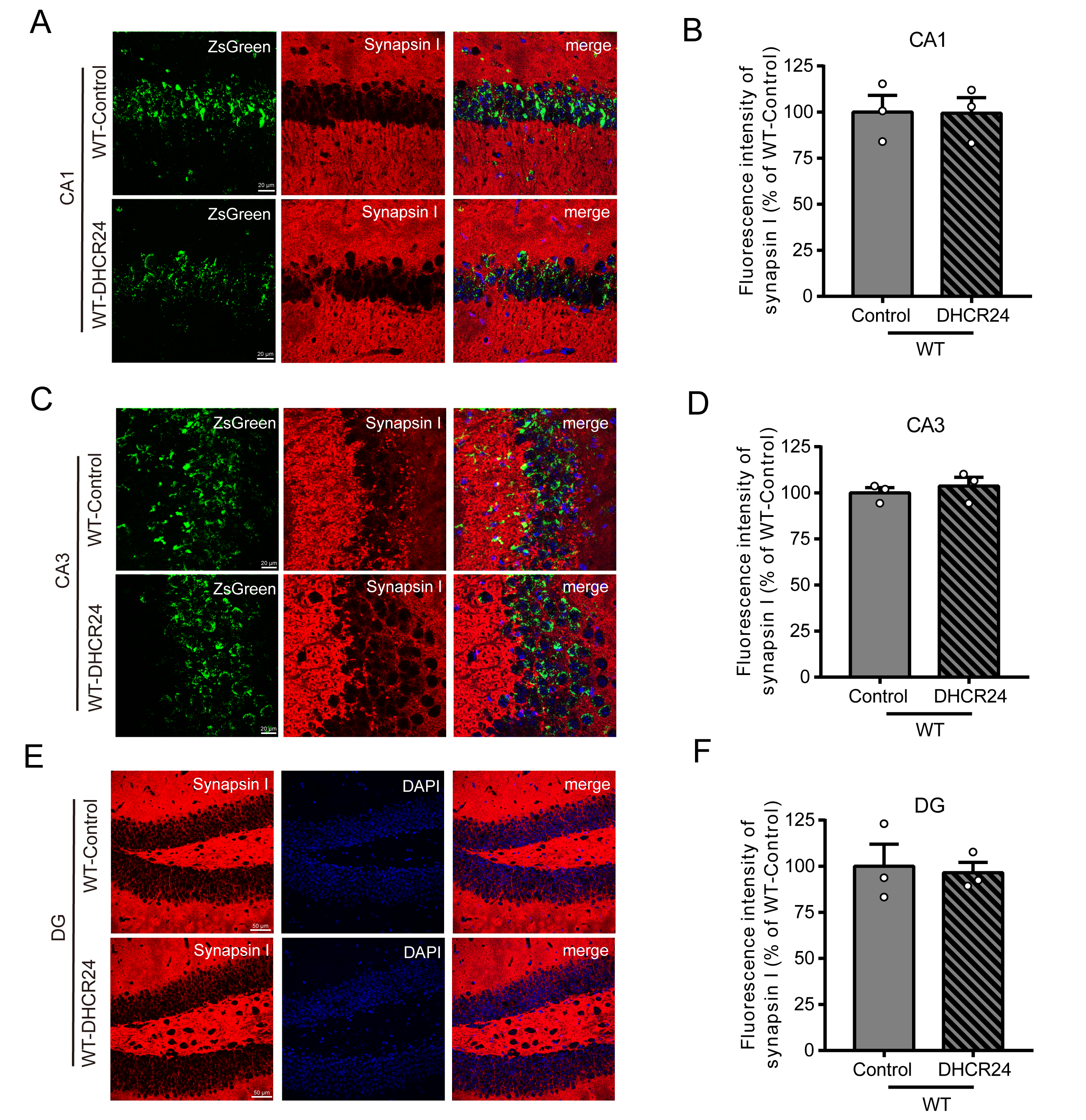

Supplement: Supplementary file 2 — Additional file 2: Fig. S2. The effect of DHCR24 knock-in on Synapsin I in the hippocampus of WT mice. A Fluorescence images of Synapsin I in the CA1 region in WT-Control group and WT-DHCR24 group. B Mean fluorescence intensity of Synapsin I in the CA1 region. C The images of Synapsin I in the CA3 region in WT-Control group and WT-DHCR24 group. D Mean fluorescence intensity of Synapsin I in the CA3 region. E Fluorescence images of Synapsin I in the DG region. F Mean fluorescence intensity of Synapsin I in the DG region. n = 3 mice per group in [A-F]. Data expressed as mean ± SEM, statistical analysis between the two groups was analyzed by unpaired two-tailed Student’s t-test. *P < 0.05; **P < 0.01; ***P < 0.001; compared with aged-matched WT-Control group [file 40478_2023_1593_MOESM2_ESM.tif]
